# Lactylation: a promising therapeutic target in ischemia-reperfusion injury management

**DOI:** 10.1038/s41420-025-02381-4

**Published:** 2025-03-13

**Authors:** Fei-xiang Wang, Guo Mu, Zi-hang Yu, Zu-an Shi, Xue-xin Li, Xin Fan, Ye Chen, Jun Zhou

**Affiliations:** 1 Department of Anesthesiology, The Affiliated Hospital, Southwest Medical University, Luzhou, Sichuan China; 2 Anesthesiology and Critical Care Medicine Key Laboratory of Luzhou, Southwest Medical University, Luzhou, Sichuan China; 3 Department of Anesthesiology, Zigong Fourth People’s Hospital, Zigong, Sichuan China; 4 Department of Anesthesiology, Fushun County People’s Hospital, Zigong, Sichuan China; 5 Department of Traditional Chinese Medicine, The Affiliated Hospital, Southwest Medical University, Luzhou, Sichuan China

**Keywords:** Cell signalling, Acute inflammation, Post-translational modifications

## Abstract

Ischemia-reperfusion injury (IRI) is a critical condition that poses a significant threat to patient safety. The production of lactate increases during the process of IRI, and lactate serves as a crucial indicator for assessing the severity of such injury. Lactylation, a newly discovered post-translational modification in 2019, is induced by lactic acid and predominantly occurs on lysine residues of histone or nonhistone proteins. Extensive studies have demonstrated the pivotal role of lactylation in the pathogenesis and progression of various diseases, including melanoma, myocardial infarction, hepatocellular carcinoma, Alzheimer’s disease, and nonalcoholic fatty liver disease. Additionally, a marked correlation between lactylation and inflammation has been observed. This article provides a comprehensive review of the mechanism underlying lactylation in IRI to establish a theoretical foundation for better understanding the interplay between lactylation and IRI.

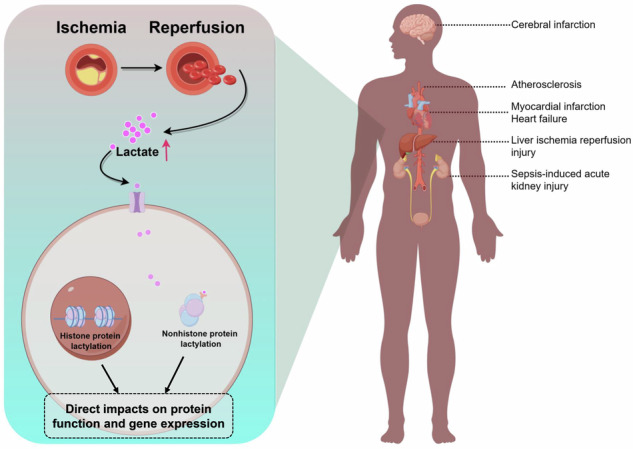

## Facts

- Lactylation can occur on lysine residues of both histones and nonhistone proteins.
- Lactylation directly regulate gene expression and protein function.
- Lactylation plays a significant role in ischemia-reperfusion injury.

## Open questions

- In the context of ischemia-reperfusion injury, which exerts a greater impact, acetylation or lactylation?
- How do different post-translational modification mechanisms influence each other?
- Is there a crosstalk between lactylation in histones and nonhistone proteins?
- How does ischemia-reperfusion injury coordinate its effects across different organs, and through what pathways?

## Introduction

Life’s sustenance hinges on a crucial equilibrium between oxygen supply and demand at the cellular level, a balance maintained through adequate blood flow perfusion [1]. Ischemia-reperfusion injury (IRI), a condition of paramount clinical significance, poses a substantial threat to patient safety [2]. Ischemia is characterized by reduced blood supply due to arterial obstruction, leading to tissue damage from inadequate nutrient and oxygen delivery. The subsequent process of reperfusion often aggravates tissue damage and promotes necrosis [3, 4]. A plethora of studies have indicated that various clinical scenarios, including sepsis, organ transplantation, shock, myocardial infarction, cerebral ischemia, and stroke, can precipitate IRI [5–10]. The effective management of systemic inflammatory response syndrome and multiple organ dysfunction syndrome, as consequences of IRI, remains a focal point in both clinical and basic research [11, 12]. Furthermore, the process of IRI involves the generation and accumulation of lactate, attributed to anaerobic glycolysis during ischemia and impaired cellular function during reperfusion [5–12]. Lactate, therefore, is an essential biomarker for gauging the severity of diseases associated with IRI. For instance, in intestinal IRI, D-lactate has been established as one of its specific diagnostic biomarkers [13].

Since its identification in 1780, lactate has been acknowledged as a metabolic byproduct generated under hypoxic conditions, known for its various adverse effects [14]. However, recent advancements, such as the lactate shuttle hypothesis, which posits lactate’s active involvement in oxidation, gluconeogenic substrates, and cellular signaling, have shifted the focus toward its broader roles beyond mere energy metabolism [15]. In 2019, the research team led by Zhao Yingming introduced the concept of lactylation, a groundbreaking revelation that highlighted lactate’s significant role in modulating histone lysine residue modifications [16]. This process offers a stark contrast to transcriptional regulation, as post-translational modifications (PTMs) like acetylation, succinylation, and malonylation can rapidly and precisely modulate protein function [17, 18]. As a novel PTM, lactylation has been extensively studied, revealing its pivotal involvement in the development and progression of various diseases, including melanoma, myocardial infarction, hepatocellular carcinoma, Alzheimer’s disease, and nonalcoholic fatty liver disease [18–24]. Additionally, a marked correlation between lactylation and inflammation has been observed [25–28].

Given the critical role of inflammation in IRI and the pronounced increase in lactate production during such injury, coupled with its diagnostic relevance in gauging disease severity, we hypothesize a significant interplay between lactylation and IRI. This review aims to provide a comprehensive analysis of the intricate relationship between lactylation and IRI, offering a solid theoretical foundation and potential research directions for IRI treatment and research.

## Current status of IRI treatment: diverse research directions, yet effective intervention strategies remain insufficient

IRI refers to the phenomenon where tissue or organ damage is unexpectedly exacerbated once the blood supply is restored after a period of ischemia. Current IRI research extensively focuses on various aspects, including oxidative stress, calcium overload, mitochondrial dysfunction, and inflammatory responses [3, 29]. To address this complex issue, researchers have explored diverse therapeutic strategies, including but not limited to remote ischemic preconditioning, remote ischemic postconditioning, and pharmacological interventions such as antioxidants, anti-inflammatory drugs, calcium channel blockers, as well as hypothermia treatment [30–32]. While these research directions and treatment methods have shown some efficacy in improving IRI outcomes, a significant gap still exists between basic research and practical clinical applications. A notable challenge is that many strategies demonstrating promising results in animal models show diminished efficacy when translated to human clinical trials. Moreover, determining the precise timing for treatment, especially the critical time point for reperfusion postconditioning, remains a major challenge that continues to perplex researchers [33].

Mitochondrial dysfunction plays a central role in the IRI process. During the ischemic phase, the rapid depletion of ATP leads to a significant decrease in mitochondrial membrane potential, which in turn triggers electron transport chain dysfunction. When blood flow is restored, the influx of oxygen causes electrons to leak from the electron transport chain and combine with oxygen molecules, producing large amounts of reactive oxygen species (ROS). Simultaneously, the opening of the mitochondrial permeability transition pore induces mitochondrial swelling and rupture, releasing apoptotic factors and ultimately leading to cell death [34, 35]. Furthermore, under hypoxic conditions, glycolysis is significantly enhanced, leading to lactate accumulation. There is a complex interplay between ROS and glycolysis, with ROS regulating the activity of key glycolytic enzymes through various pathways, further promoting lactate production [28, 36]. As an important substrate for lactylation, the increase in lactate concentration significantly enhances the level of lactylation and has a crucial impact on the final outcome of IRI.

PTMs play a vital role in biological systems as a mechanism for rapid and precise regulation of protein function. Lactylation, a recently discovered important form of PTM, plays a key role in various IRI-related diseases, such as myocardial infarction, septic shock, and cerebral IRI [20, 37, 38]. Given that lactylation has rapid, precise, and energy-efficient regulatory characteristics and is closely related to key mitochondrial damage in IRI, an in-depth study of the connection between lactylation and IRI will help us better understand the pathological mechanisms of IRI and potentially discover new therapeutic targets.

## Lactate metabolism

### Sources of lactate and its role as a signaling molecule

**Fig. 1:**
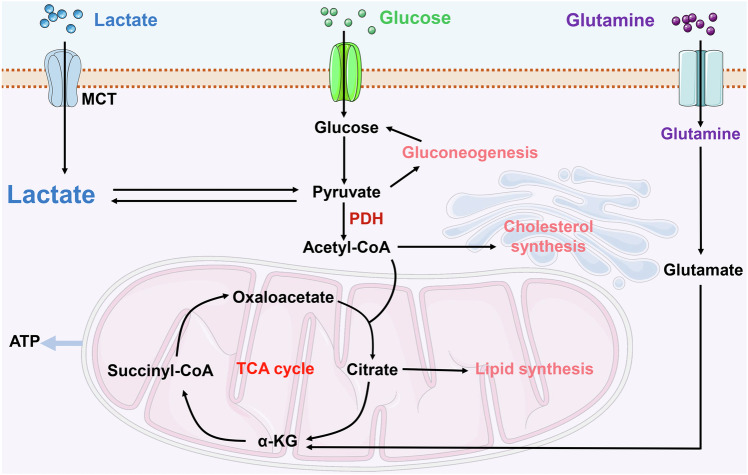
Overview of lactate metabolism. Lactate production and metabolism within cells encompass several key steps: Initially, lactate is translocated across the cellular membrane via the MCT, moving from the extracellular environment into the cytoplasm. Lactate formation occurs either through glycolytic processes or from the breakdown of glutamine. Cellular lactate catabolism is divided into two primary pathways. The first involves the oxidation of lactate to pyruvate, which is then transported into the mitochondria for subsequent catabolism within the TCA cycle. The second pathway involves the conversion of lactate back to glucose via gluconeogenesis. α-KG α-ketoglutaric acid, MCT monocarboxylate transporter, PDH pyruvate dehydrogenase, TCA tricarboxylic acid. Created with Figdraw.

Lactate plays a pivotal role in energy metabolism regulation and signal transduction, acting as a key cellular fuel source, particularly during intense physical activity and infection, when an imbalance exists between oxygen supply and ATP demand [15]. Under conditions of limited cellular oxygen availability, such as hypoxia or anaerobic environments, mitochondrial oxidative phosphorylation is hindered, leading to suboptimal pyruvate utilization. As a result, cells predominantly rely on glycolysis for energy production, culminating in lactate formation. Lactate is primarily synthesized during glycolysis as a common byproduct of glucose metabolism. In anaerobic or hypoxic conditions, glucose is metabolized into two molecules of pyruvate within the cytoplasm [14]. However, the lack of sufficient oxygen for electron acceptance in the electron transport chain prevents pyruvate from entering the mitochondria for further oxidation. At this juncture, lactate dehydrogenase facilitates the conversion of pyruvate into lactate, simultaneously replenishing NAD^+^ levels, thereby enabling the continuation of glycolysis [14, 15] (Fig. 1).

The elucidation of the Warburg effect has significantly advanced our comprehension of lactate metabolism [16]. This phenomenon demonstrates that, even in the presence of oxygen, tumor cells predominantly utilize glycolysis over mitochondrial oxidative phosphorylation for energy production [39]. The Warburg effect facilitates the rapid proliferation of tumor cells in an oxygen-rich milieu by primarily depending on glycolysis for energy. Moreover, it contributes to tumor growth by supplying necessary growth factors and aids in evading immune detection [16, 39].

Monocarboxylate transporters (MCTs) are a family of solute carrier proteins chiefly responsible for the movement of one-carbon metabolites, such as lactate and keto acids, between cells. In humans, the MCTs encompasses several members, with MCT1 and MCT4 being the most thoroughly researched [40]. These transporters operate via a proton-linked mechanism, allowing lactate molecules to cross the cell membrane in tandem with protons (H^+^). This process maintains a dynamic equilibrium, regulating the concentration gradients of lactate and protons. Typically, MCTs facilitate passive transport, enabling lactate to move in or out of cells based on the direction of the concentration gradient [15, 40]. These transporters are instrumental in maintaining the balance of lactate levels both inside and outside the cell, especially in hyperglycolytic conditions such as those found in tumor cells [40]. Investigating these transporters not only deepens our understanding of regulatory mechanisms in cellular metabolism but also presents potential therapeutic avenues for certain diseases.

Currently, lactate fulfills three key roles: it acts as the primary energy source, serves as a significant precursor for gluconeogenesis, and functions as signaling molecules with autocrine, paracrine, and endocrine-like actions, collectively referred to as “lactormone” [41]. For example, recent studies have demonstrated the multifaceted role of lactate in various physiological and pathological processes. Notably, lactate has been shown to induce hypoxic responses, while inhibition of intracellular lactate production correspondingly suppresses these responses [42]. Furthermore, mitochondrial antiviral signaling protein has been identified as a direct sensor of lactate, thus establishing a link between energy metabolism and innate immunity [43]. Intriguingly, the continuous accumulation of lactate promotes the remodeling of abnormal late-promoting complexes and overcomes the effects of antimitotic drugs through mitotic slippage mechanisms, thereby regulating cell cycle progression and proliferation [44]. These findings collectively underscore the critical role of lactate in diverse pathophysiological processes.

The synthesis of lactate is an adaptive biological response, allowing cells to sustain energy metabolism via distinct pathways under aerobic and hypoxic conditions. In an aerobic environment, the generation of lactate aids in maintaining the equilibrium between glycolysis and mitochondrial metabolism. Conversely, under hypoxic conditions, the production of lactate becomes essential to facilitate the continuous operation of glycolysis.

### Lactate regulates immune cells and inflammatory responses

**Fig. 2:**
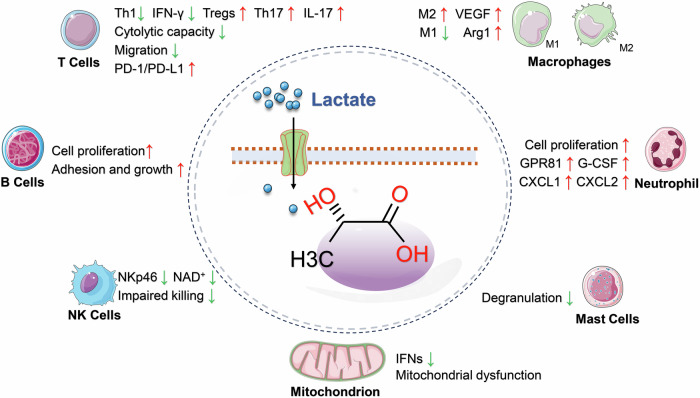
Lactate regulates immune cell behavior to impact inflammation. Lactate plays a significant role in cellular metabolism by inhibiting mitochondrial oxidative phosphorylation, thereby contributing to mitochondrial dysfunction. Moreover, lactate demonstrates the capacity to modulate the activation of immune cells such as T cells, B cells, NK cells, neutrophils, macrophages, and mast cells. This modulation leads to the secretion of specific cytokines, which exert a profound influence on the inflammatory response. Arg1 arginase-1, CXCL CXC motif chemokine, G-CSF granulocyte colony-stimulating factor, GPR81 G-protein-coupled receptor 81, IFN-γ interferon-γ, IFNs type I interferons, IL-17 interleukin-17, NKp46 lysis receptor 46 of natural killer cell, PD-1 programmed cell death protein 1, PD-L1 programmed cell death protein 1 ligand, Th1 type 1 helper T cells, Th17 type 17 helper T cells, Tregs regulatory T cells, VEGF vascular endothelial growth factor. Created with Figdraw.

During inflammation, the heightened energy requirements of immune cells (such as macrophages and T cells) are predominantly met through an increase in the glycolytic process, leading to significant lactate production [45]. These metabolic shifts not only supply essential energy to immune cells but may also modulate the inflammatory response via lactate or its derivatives. Lactate functions not merely as a metabolite but also as a signaling molecule, influencing inflammation by regulating immune cell behavior [46]. Additionally, lactate accumulation contributes to the acidification of the local microenvironment, potentially impacting immune cell functions, including T-cell activity and cytokine production [45] (Fig. 2).

Lactate’s role varies with the metabolic state and specific cell type under investigation, impacting various pathological processes. It influences signaling pathways through mechanisms such as PTMs, interaction with G-protein-coupled receptors, and activation of transcription factors like NF-κB and HIF1. These interactions regulate the expression of cytokines, chemokines, adhesion molecules, and enzymes integral to immune responses and metabolic processes [47]. Moreover, lactate is intimately associated with the activation states of various immune cells, including monocytes, natural killer cells, mast cells, T cells, tumor cells, fibroblasts, and macrophages. In response to harmful stimuli, lactate can modulate the differentiation processes of Th1, Th17, and regulatory T cells, which are essential for maintaining immune homeostasis [48]. These insights imply that targeting lactate regulation could be a promising therapeutic approach for treating inflammatory diseases.

### Targeting lactate and related inflammatory responses to improve IRI prognosis

**Fig. 3:**
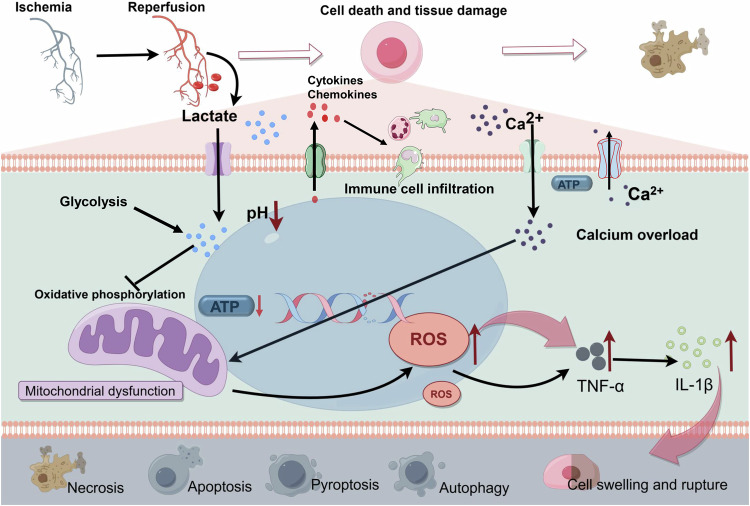
Simplified schematic representation of ischemia-reperfusion injury. In the context of ischemia-reperfusion injury, there is a reduction in intracellular ATP levels and pH, attributed to anaerobic metabolism and the accumulation of lactate. Consequently, there is a compromise in the ATP-dependent ion transport mechanism. This disruption precipitates intracellular and intra-mitochondrial calcium overload, leading to mitochondrial dysfunction and a subsequent upsurge in the production of inflammatory factors. Additionally, the release of the corresponding cytokines and chemokines induces immune cell infiltration. These biochemical alterations result in cellular swelling, rupture, and demise. Modes of cell death encompass necrosis, apoptosis, pyroptosis, autophagy, and other associated pathways. IL-1β interleukin-1β, ROS reactive oxygen species, TNF-α tumor necrosis factor-α. Created with Figdraw.

IRI is critical in the pathogenesis and advancement of many diseases. During ischemia, cellular ATP production through oxidative phosphorylation is compromised due to a restricted oxygen supply. This limitation forces cells to depend on anaerobic metabolism for lactate generation. Subsequently, lactate accumulation causes a decrease in intracellular pH, negatively impacting cellular functionality and potentially inducing cell death [41]. Prolonged ischemic conditions intensify this metabolic disturbance by further reducing ATP levels and intracellular pH, a result of sustained anaerobic metabolism and lactate accumulation. This leads to a dysfunction in ATP-dependent ion transport processes, culminating in increased intracellular and mitochondrial calcium levels (calcium overload), cellular swelling, rupture, and cell death via various pathways including necrosis, apoptosis, and autophagy. Despite reperfusion reestablishing oxygenation, it paradoxically exacerbates the injury by promoting reactive oxygen species production and encouraging pro-inflammatory neutrophils to infiltrate the ischemic tissues, thereby amplifying the ischemic damage [49] (Fig. 3).

IRI is known to initiate both local and systemic inflammatory responses. During the ischemic phase, cells undergoing stress release a multitude of inflammatory mediators, including cytokines and chemokines. These mediators further stimulate immune cell activation during reperfusion [49]. In this phase, the resultant cellular injury and death precipitate the secretion of various inflammatory agents (e.g., tumor necrosis factor-α, interleukins) and chemoattractants, leading to significant tissue inflammation and damage [50]. This inflammatory cascade increases vascular permeability, resulting in the leakage of plasma components into adjacent tissues, which contributes to tissue swelling and further aggravates the damage [51]. Upon reestablishment of blood flow following prolonged ischemia, the local inflammatory response and reactive oxygen species production intensify, thereby exacerbating secondary tissue injury. The cellular damage caused by chronic IRI can culminate in various forms of cell death, including apoptosis, autophagy, necrosis, and necroptosis [3].

The management of lactate levels and inflammatory processes is crucial in the development and progression of IRI. Consequently, targeting these aspects not only diminishes the severity of IRI but also provides innovative strategies for treating related diseases. For instance, postconditioning with lactate-enriched blood has been shown to mitigate fatal human reperfusion injury, highlighting the potential of lactate-based interventions in improving IRI outcomes [52]. The integration of these insights into clinical practice promises to improve therapeutic outcomes and enhance patient quality of life.

## Lactylation - a novel PTM with lactate as the substrate

**Fig. 4:**
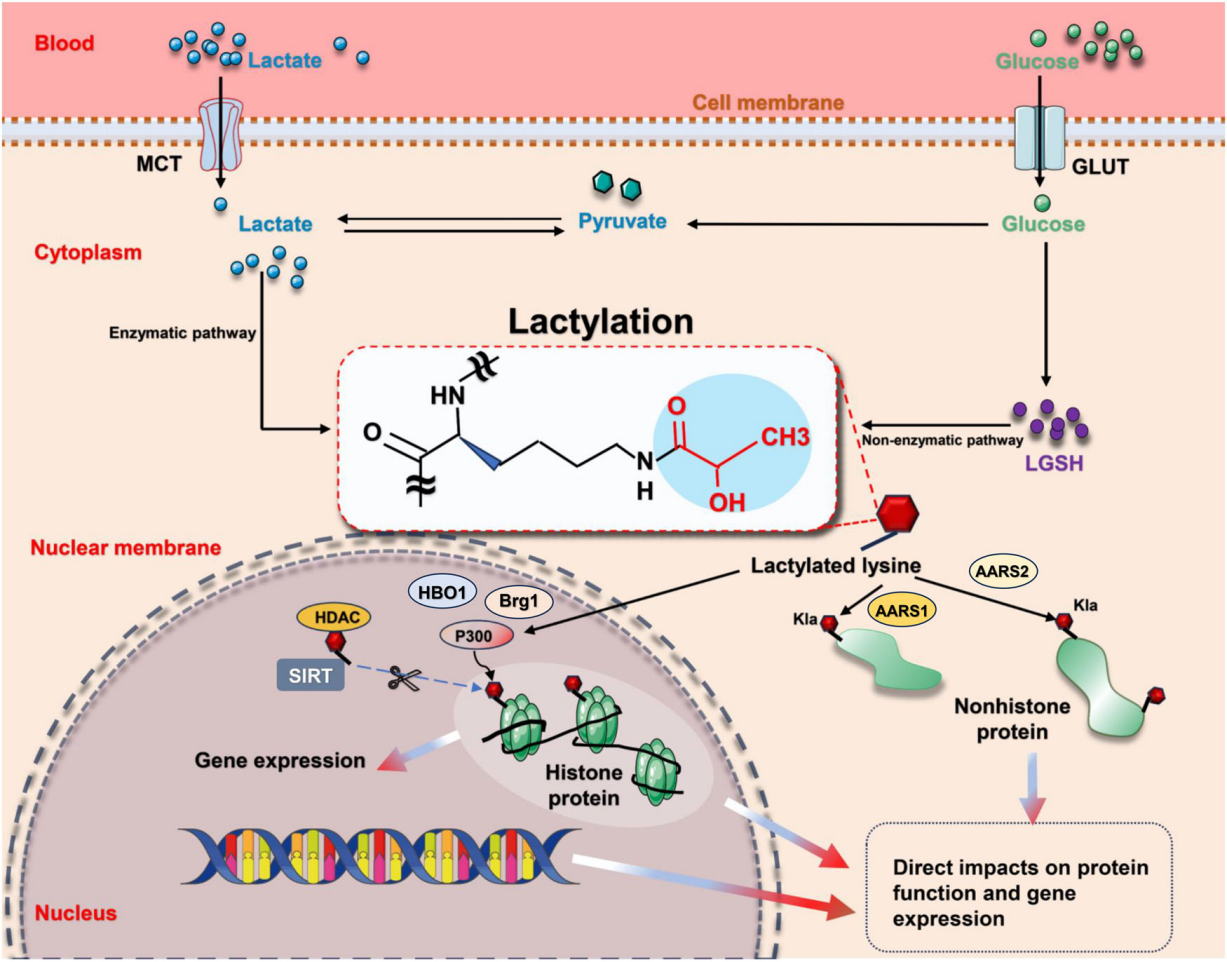
Overview of the lactylation mechanism. Lactate transport from extracellular to intracellular compartments, coupled with its generation via glycolysis, facilitates the lactylation of lysine residues on both histone and nonhistone proteins. This modification occurs through either enzyme-dependent or enzyme-independent pathways. Such a process directly influences protein function and plays a crucial role in the regulation of gene expression. AARS mitochondrial alanyl-tRNA synthetase, Brg1 brahma-related gene 1, GLUT glucose transporter, HBO1 histone acetyltransferase binding to ORC-1, HDAC histone deacetylase, Kla lysine lactylation, LGSH lactyl-glutathione, MCT monocarboxylate transporter, P300 acetyltransferase p300, SIRT histone deacetylase sirtuin. Created with Figdraw.

PTMs profoundly affect almost all proteins, playing a vital role in preserving the functionality and diversity of the proteome. Recent research has unveiled that lactate can facilitate the lysine lactylation (Kla) of core histone proteins, thereby modulating gene expression (e.g., Arg1) and influencing the pathogenesis of various conditions, such as tumor formation [16, 21] (Fig. 4). Subsequent studies have extended these findings to nonhistone proteins, exemplified by modifications in high mobility group box-1 (HMGB1) [28]. Lactylation has emerged as a pivotal factor in a spectrum of diseases, encompassing inflammation, multiple cancers, cognitive disorders, sepsis, and more [21, 23, 28, 41].

Since its discovery in 2019, lactylation has rapidly emerged as a promising research direction [16]. Initial investigations primarily focused on histone modifications, particularly at key sites such as H3K9, H3K18, H4K5, H4K8, and H4K12. The pivotal role of these sites in the pathogenesis of related diseases has been extensively validated through diverse methodologies [16, 53, 54]. With the advent of advanced technologies such as deep sequencing, the scope of lactylation research has expanded to encompass nonhistone proteins. This includes various proteins such as HMGB1, METTL3, PKM2, and AK2, and spans a wide range of diseases including sepsis, liver IRI, colon cancer, skin wounds, and hepatocellular carcinoma [21, 28, 55, 56]. Current research trends indicate parallel development in both histone and nonhistone lactylation, with an increasing focus on the latter. A growing body of evidence highlights the crucial role of lactylation in various diseases, particularly in tumor-related and IRI-related conditions associated with elevated lactate levels [18–22].

Moreover, there is a close interplay between lactylation and inflammatory mediators. A study on spinal cord injury demonstrated that the introduction of lactate significantly augments lactylation levels in microglia, effectively inhibiting the expression of pro-inflammatory mediators such as IL-1β, iNOS, and TNF-α [57]. Corroborating these findings, another study on experimental autoimmune uveitis (EAU) revealed enhanced lactate modification of Yin Yang-1 (YY1) in retinal microglia. Notably, YY1 lactylation not only promoted microglial activation but also enhanced their proliferation and migration capabilities. Inhibition of lactylation significantly suppressed microglial activation in EAU, thereby attenuating the inflammatory response [58]. These findings collectively underscore the significant role of lactylation in regulating inflammatory mediators and its involvement in disease progression. However, there is currently a paucity of research investigating whether inflammatory mediators can, in turn, modulate lactylation levels. Further studies are warranted to elucidate the potential regulatory effects of inflammatory mediators on lactylation.

Overall, since its initial identification in 2019, research on protein lactylation has evolved through several distinct developmental phases. Initial investigations predominantly focused on histone modifications, particularly at H3K9 and H3K18 sites, establishing their fundamental roles in transcriptional regulation [16, 53, 54]. Subsequently, the research scope expanded significantly to encompass nonhistone proteins, unveiling complex cellular regulatory networks. Contemporary literature analysis has revealed three major trends in lactylation research: First, lactylation modifications exert predominant effects on proteins involved in metabolic regulation and inflammatory responses, as demonstrated by: PKM2’s regulatory role in macrophage metabolism [56], HMGB1’s critical function in inflammatory responses [28], AK2’s essential involvement in cellular energy homeostasis [21]; Second, substantial crosstalk exists between lactylation and other PTMs, particularly acetylation, as evidenced by: The dual regulatory functionality of HDACs [59, 60], The dynamic equilibrium between lactylation and acetylation modifications of macrophage HMGB1 [28]; Third, lactylation modifications exhibit distinct tissue-specific distribution patterns, with notably higher prevalence in metabolically active tissues, as revealed by comprehensive proteomic analyses demonstrating elevated occurrence in: liver kupffer cells [61], cardiac tissue [62] and tumor microenvironment [22]. Given lactate’s pivotal role in energy metabolism and signal transduction, systematic investigation of both histone and nonhistone lactylation modifications has become essential. These investigations are particularly crucial for enhancing our understanding of the molecular mechanisms underlying inflammatory and metabolic disease development.

### Histone lactylation

Histones, alkaline proteins located within the chromatin of eukaryotic cells, form nucleosome structures with DNA and are integral to chromatin architecture [63]. Histones undergo various PTMs, such as acetylation, malonylation, and succinylation. These modifications can alter the spatial structure of proteins, thereby regulating a wide array of cellular physiological and biochemical processes [64]. The study by Zhang et al. [16] revealed that lactate induces histone lysine lactylation, influencing gene transcription in a dose-dependent manner. Furthermore, they identified the enzyme acetyltransferase p300(p300), a renowned histone acetyltransferase that is recruited by P53, as responsible for modifying histone Kla [16, 18]. In a significant advancement in 2022, Zhang et al. delineated the role of Class I histone deacetylases (HDAC1–3) and histone deacetylase sirtuins (SIRT1–3) as delactylases in vitro. This discovery bridged a critical gap in delactylation research, integrating the novel lactylation PTM into a cohesive biochemical process [18, 59].

Additionally, lactyl-CoA, identified in mammalian cells and tissues via liquid chromatography-mass spectrometry, acts as the substrate for enzymatic lactylation. Non-enzymatic lactylation also occurs, involving the transfer of the lactyl group by lactyl-glutathione. These two distinct mechanisms can independently or jointly contribute to lactylation [22]. The modification of lysine residues through lactylation and acetylation significantly impacts the levels of metabolites such as lactate, acetyl-CoA, and pyruvic acid, as well as their intricate interplay. These metabolic alterations have profound implications for subsequent cellular pathological and physiological processes and revealed the potential correlation between lactylation and acetylation, given their similar mechanisms involving writers, erasers, and readers [18, 60].

Currently, the roles of Kla sites on five histone proteins in various diseases have been elucidated, with particular emphasis on the K18 site of histone 3 (H3K18). This site has been the subject of intensive research in human, rat, and mouse models, as summarized in Table 1. Histone lactylation has been predominantly identified in tumor-associated diseases, including lung cancer, melanoma, breast cancer, prostate cancer, colon cancer, renal cell carcinoma, colorectal cancer, and bladder cancer. Tumors are known to undergo metabolic reprogramming, tumor-associated inflammation, evasion of immunosurveillance, and genome instability. These changes facilitate sustained proliferation, resistance to apoptosis, evasion of growth suppressors, replicative immortality, tumor angiogenesis, and invasion and metastasis [22]. The Warburg effect, characterized by aerobic glycolysis, is closely linked to the onset and progression of tumors and promotes lactate production. This underscores the interconnection between lactylation and tumor biology, as evidenced by extensive research [16, 22]. For example, histone lactylation has been shown to drive oncogenesis by enhancing m6A reader protein YTH N6-methyladenosine RNA-binding protein 2 (YTHDF2) expression in ocular melanoma [19].

**Table 1:** Overview of identified lactylation sites in histone and nonhistone proteins and their association with diverse pathological conditions.

Lactylation	Sites	Species	Diseases	References
Histone lactylation	H2BK5/H3K18/H3K23/H4K5/H4K8/H4K12	Mouse	Lung cancer/Melanoma	[16]
	H4K12	Human/Mouse	Alzheimer’s disease	[23]
	H3K18	Rat	Cerebral ischemia	[38]
	H3K9/H3K56	Human/Mouse	Hepatocellular carcinoma	[54]
	H1	Mouse	Stress	[131]
	H3K18	Human	Preeclampsia/Endometriosis/Breast cancer/Uveal melanoma/Malignant pleural effusion/Septic Shock	[37, 65, 66, 132–134]
	H3K18	Mouse	Myocardial infarction/Alzheimer’s disease/Intestinal inflammation/Cellular reprogramming/Ulcerative Colitis/Arsenite-related idiopathic pulmonary fibrosis/Cognitive dysfunction/Sepsis/Prostate cancer	[20, 67, 68, 135–140]
	H3K18	Human/Mouse	Ocular melanoma/Colon cancer/Renal cell carcinoma/Liver fibrosis/Prostate cancer/Lung cancer/Colorectal cancer/Bladder cancer/Osteoporosis/Glioblastoma/Colorectal cancer liver metastases	[19, 53, 73, 141–147]
	H3K18/H4K5/H4K8/H4K12	Human	Acute myeloid leukemia	[148]
	H3K18/H4K5	Rat	Pulmonary hypertension	[149]
	H3K14/H4K12	Rat/Mouse	Toxoplasma gondii	[150]
	H3K18/H3k23	Mouse	Embryonic development	[151]
	H4K8/H4K5	Human	Colon cancer	[152]
	H4K12	Mouse	Anaplastic thyroid cancer	[153]
	K56 of H3.3/K49 of H2B/K10,14 of H2AZ/K8,18,53 of H2BZ	Human/P. falciparum	Malaria	[154]
	H3K9	Mouse	Muscle regeneration	[155]
	H3K18/H4K12	Human/Mouse	Decidualization	[156]
	H3K18	Holstein cows	Mammitis	[157]
	H3K9/H3K18	Mouse	Schizophrenia	[158]
	K6 of H2B/K80 of H4	Human/Rat	Dyslipidaemia	[159]
	H3K9/H3K14	Mouse	Hepatocellular carcinoma	[160]
	H3K18/H4K12	Mouse	Type 2 Diabetes	[161]
	H4K12	Human/Mouse	Lung cancer brain metastasis	[162]
	H3K14/H3K18	Human	Lung adenocarcinoma	[163]
Nonhistone lactylation	K28 of AK2	Human	Hepatocellular carcinoma	[21]
	K673 of FAS	Human/Mouse	Nonalcoholic fatty liver disease	[24]
	HMGB1	Mouse	Sepsis/Liver ischemia-reperfusion injury	[28, 83]
	K281/K345 of METTL3	Human/Mouse	Colon cancer	[55]
	K62 of PKM2	Mouse	Skin wound	[56]
	CCR8	Human/Mouse	Glioblastoma	[73]
	Snail1	Mouse	Myocardial infarction	[82]
	K72 of MOESIN	Human/Mouse	Hepatocellular carcinoma	[76]
	K183 of Yin Yang-1	Human/Mouse	Retinopathy of prematurity	[121]
	K1897 of α-MHC	Human/mouse	Heart failure	[62]
	K356/K781 of Vps34	Human/Mouse	Lung cancer /Gastric cancer	[74]
	HIF1α	Human	Prostate cancer	[70]
	K229 of METTL16	Human/Mouse	Gastric cancer	[71]
	MRE11	Human/Mouse	Colon cancer	[164]
	USP14/ABCF1	Human	Hepatocellular carcinoma	[77]
	K356/K781 of VPS34	Human/Mouse	Autophagy	[165]
	K40 of α-tubulin	Mouse	Cytoskeleton function	[166]
	K20 of Fis1	Mouse	Acute kidney injury	[72]
	β-catenin	Human/Mouse	Colorectal cancer	[75]
	FASN	Mouse	High-intensity interval training	[167]
	K348 of CCNE2	Human/Mouse	Hepatocellular carcinoma	[78]
	K271 of Mecp2	Mouse	Atherosclerosis	[168]
	IDH3G	Mouse	Lung Adenocarcinoma	[119]
	C-myc	Liver cancer cell	Hepatocellular carcinoma	[169]
	LCP1	Rat	Cerebral infarction	[118]
	K124 of CENPA	Human/Mouse	Hepatocellular carcinoma	[170]
	Sox10	Human/Mouse	Neointimal hyperplasia	[171]
	METTL3	Mouse	Intracerebral hemorrhage	[112]

*α-MHC* α-myosin heavy chain, *ABCF1* ATP-binding cassette subfamily F member 1, *AK2* adenylate kinase 2, *CCNE2* cyclin E2, *CCR8* chemokine receptor 8, *CENPA* centromere protein A, *C-myc* a small family of proto-oncogenes, *FASN* fatty acid synthase, *Fis1* mitochondrial fission 1 protein, *HIF1α* hypoxia-inducible factor 1α, *HMGB1* high mobility group box-1, *IDH3G* isocitrate dehydrogenase 3 gamma, *LCP1* lymphocyte cytosolic protein 1, *MECP2* methyl CpG binding protein 2, *METTL3* methyltransferase-like 3, *METTL16* methyltransferase-like 16, *MRE11* a crucial homologous recombination protein, *PIK3C3/VPS34* phosphatidylinositol 3-kinase catalytic subunit type 3, *PKM2* pyruvate kinase M2, *SOX10* high mobility group box gene 10, *USP14* ubiquitin-specific protease 14, *Vps34* vacuolar protein sorting 34.

Beyond its significant correlation with cancer, histone lactylation is also implicated in a range of diseases, including preeclampsia, endometriosis, septic shock, myocardial infarction, Alzheimer’s disease, and various intestinal disorders characterized by inflammation [20, 37, 65–68]. These associations underscore the role of lactate as an extracellular energy substrate in epigenetic regulation, particularly through histone lactylation. During ischemic episodes, where oxygen deprivation leads to anaerobic glycolysis, there is a notable increase in lactate production, resulting in enhanced lactylation. The effectiveness of therapeutic strategies targeting histone lactylation sites in mitigating IRI and facilitating the recovery of associated functions has been validated in several studies [20, 37, 38]. For example, recent studies have elucidated the pivotal role of histone lactylation in regulating the dual functions of monocyte-macrophages, specifically their anti-inflammatory and pro-angiogenic properties, in the context of myocardial infarction (MI). This process is mediated through the promotion of repair gene transcription, resulting in improved cardiac repair and function post-MI. Notably, lactylation at the H3K18 site plays a central role in this physiological process [20]. Furthermore, lactylated histone H3K18 shows promise as a potential biomarker for assessing the severity and predicting infectious shock, while also regulating the anti-inflammatory function of macrophages [37]. Studies on cerebral IRI have revealed that lactate dehydrogenase A mediates histone lactylation induced by cerebral IRI by targeting HMGB1, thereby triggering pyroptosis [38]. These findings provide new insights into the complex role of lactylation in various physiological and pathological processes associated with cerebral IRI.

Systematic analysis of current evidence has revealed several critical aspects regarding histone lactylation modification. While extensively studied modification sites such as H3K18 have been well-characterized across various pathological conditions [20, 37, 65], investigation of other histone sites remains insufficient. Although this site-specific investigative approach has yielded valuable insights, it potentially limits comprehensive understanding of the global impact of histone lactylation modifications. The temporal dynamics of histone lactylation modifications warrant particular attention, as exemplified in myocardial infarction, where H3K18 lactylation demonstrates distinct patterns during various phases of cardiac repair [20]. However, comparable temporal analyses in other disease contexts remain limited, particularly in IRI, where intervention timing is crucial for therapeutic efficacy. Moreover, current research faces several methodological challenges: 1. Excessive reliance on correlative analyses between specific histone site lactylation levels and disease outcomes, with insufficient investigation of causal relationships; 2. Predominant focus on endpoint measurements rather than temporal trajectory analyses; 3. Limited understanding of tissue-specific variations in histone lactylation patterns, suggesting complex regulatory mechanisms requiring further investigation. However, given the fundamental role of energy metabolism in biological processes and current research evidence, investigations focusing on lactate and histone lactylation modifications represent promising research directions for developing therapeutic strategies against metabolic diseases, particularly malignancies.

### Nonhistone lactylation

Owing to technological advancements, notably the advent of liquid chromatography-mass spectrometry, the research focus has expanded from histone lactylation to encompass nonhistone lactylation [55, 56]. A 2020 study revealed that proteomic analysis identified 273 lactylation sites across 166 proteins within Botrytis cinerea, a harmful necrotrophic fungal pathogen. Intriguingly, nearly two-thirds of these lactylated proteins were observed to be cytoplasmic [69]. Sung et al.’s investigation into liver Kupffer cells disclosed 298 lactylation sites in 181 proteins, predominantly localized in the nucleus (60%), cytoplasm (28%), mitochondria (4%), cell projections (3%), and chromosomes (3%) [61]. These findings suggest that, beyond histone lactylation, a vast array of nonhistone proteins are capable of undergoing lactylation. Thus, advancing research is expected to progressively uncover the complex relationship between nonhistone lactylation and the development and progression of diseases.

The current research on lactylation in nonhistone proteins predominantly focuses on its implications in tumors, liver and kidney injuries, as well as heart failure [21, 24, 62, 70–72]. Similar to histone lactylation, nonhistone lactylation’s correlation with various cancer-related diseases has been established, encompassing colon cancer, hepatocellular carcinoma, lung cancer, gastric cancer, prostate cancer, colorectal cancer, and glioblastoma [21, 55, 70, 71, 73–75]. Crucial in tumor immune escape, tumor-infiltrating myeloid cells have been shown to be significantly affected by lactylation. Recent studies have identified that lactylation at K281 and K345 sites of methyltransferase-like 3(METTL3) can effectively modulate the epigenetic modification of its associated RNA N^6^-methyladenosine, thereby inducing immunosuppression in these cells [55]. Additionally, the liver, as the primary metabolic organ for lactate, has been the subject of numerous studies highlighting lactate accumulation-mediated lactylation in liver tumors [21, 76–78]. For instance, lactylation of adenylate kinase 2 at the K28 site has been found to inhibit its function, thereby facilitating proliferation and metastasis in hepatitis B virus-related hepatocellular carcinoma cells [21].

Notably, recent research has identified novel nonhistone lactate transferases, expanding our understanding of lactylation mechanisms. Mitochondrial alanyl-tRNA synthetase 1 (AARS1) demonstrates a strong affinity for lactate and catalyzes the formation of lactate-AMP, subsequently transferring lactate to lysine receptor residues. This process induces lactylation of P53 and promotes tumor development by regulating mechanisms such as the Hippo pathway [79, 80]. Similarly, AARS2 has been found to regulate cell metabolism through lactylation, specifically by limiting the influx of acetyl-CoA in pyruvate and fatty acid oxidation processes, ultimately inhibiting oxidative phosphorylation [81]. These findings greatly enrich our understanding of the research field of nonhistone “writers.”

Additionally, a significant link has been established between nonhistone lactylation and various non-neoplastic diseases. Pyruvate kinase M2, a critical regulator of metabolic adaptations in pro-inflammatory macrophages, has been a key focus in this area. Research by Wang et al. revealed that increased lactylation levels of pyruvate kinase M2 at the K62 site due to lactate exposure inhibits its transition from tetramer to dimer. This inhibition enhances its pyruvate kinase activity and decreases its nuclear distribution, consequently facilitating the transition of pro-inflammatory macrophages to a reparative phenotype [56].

It is worth noting that there is a significant association between nonhistone lactylation and IRI. Research has revealed that endothelial-mesenchymal transition plays a central role in the pathological process of cardiac fibrosis. Specifically, lactate can accelerate the endothelial-mesenchymal transition process after myocardial infarction, thereby exacerbating cardiac fibrosis and worsening cardiac function, with Snail1 lactylation playing a key role in this complex mechanism [82]. Additionally, in the field of heart failure research, lactylation of α-myosin heavy chain has been observed to exhibit dynamic regulation characteristics during the progression of heart failure. This modification is not only an important determinant of overall cardiac structure and function but also provides us with a new approach to alleviate heart failure symptoms - by enhancing the lactylation level at the K1897 site of α-myosin heavy chain [62]. Similarly, in the context of liver IRI, nonhistone lactylation also exhibits unique biological effects. Studies have shown that heat shock protein A12A (HSPA12A) in hepatocytes can effectively alleviate liver IRI pathological damage by inhibiting glycolysis-mediated HMGB1 lactylation and hepatocyte secretory activity, thereby weakening the chemotaxis and activation ability of macrophages. This finding suggests that targeted therapy for hepatocyte HSPA12A may show potential in future clinical treatment of liver IRI [83].

Overall, accumulated evidence has elucidated multiple mechanisms underlying nonhistone lactylation regulation. First, metabolic regulation occurs through direct modification of metabolic enzymes affecting their activity and stability, as demonstrated in the glycolysis pathway regulation [56]. Second, transcriptional regulation involves lactylation modification of transcription factors that modulate gene expression programs, particularly evident in inflammatory responses [28]. Third, structural regulation encompasses modifications of structural proteins that influence cellular architecture and function, as exemplified by cardiac myosin modifications [62]. In the context of IRI, several distinctive patterns have emerged: cell-type specificity, where different cell populations exhibit distinct lactylation responses, notably demonstrated in hepatocyte HSPA12A-mediated regulation of HMGB1 lactylation [83]; temporal dynamics, where lactylation modification patterns demonstrate dynamic alterations during various stages of injury and repair [82]; and therapeutic implications, where targeting lactylation modifications shows therapeutic potential [62, 83], although clinical translation remains challenging.

Furthermore, current research faces several significant limitations in the field of protein lactylation. A primary technical constraint involves the detection and quantification of in vivo lactylation modifications. Sun et al. noted that current mass spectrometry-based analytical methods can identify only a subset of lactylation sites, potentially overlooking low-abundance modifications [61]. Additionally, understanding of tissue-specific regulatory patterns remains insufficient. Although lactylation sites have been identified across various tissues, comprehension of tissue-specific regulatory mechanisms remains limited. For instance, Hong et al.’s comprehensive analysis of hepatocellular carcinoma revealed unique lactylation patterns, though the underlying regulatory mechanisms remain incompletely understood [77]. Similarly, Sun et al.’s investigations of Kupffer cells demonstrated distinct cellular lactylation profiles [61], revealing notable discrepancies with hepatocellular findings [77], thus necessitating further investigation. Furthermore, functional impacts of lactylation modifications demonstrate significant context dependency under different pathological conditions. Yang et al.’s research demonstrated that lactylation promotes metabolic adaptation in hepatocellular carcinoma [21], while Xiong et al.’s study revealed lactylation’s involvement in immunosuppression mediated by tumor-infiltrating myeloid cells [55]. These apparently contradictory findings underscore the necessity for careful evaluation of context-specific functions when developing therapeutic strategies. Moreover, while some studies indicate beneficial effects of lactylation under certain conditions [20], others suggest potential adverse effects [28], emphasizing the importance of comprehensive investigation to understand these contrasting phenomena.

**Fig. 5:**
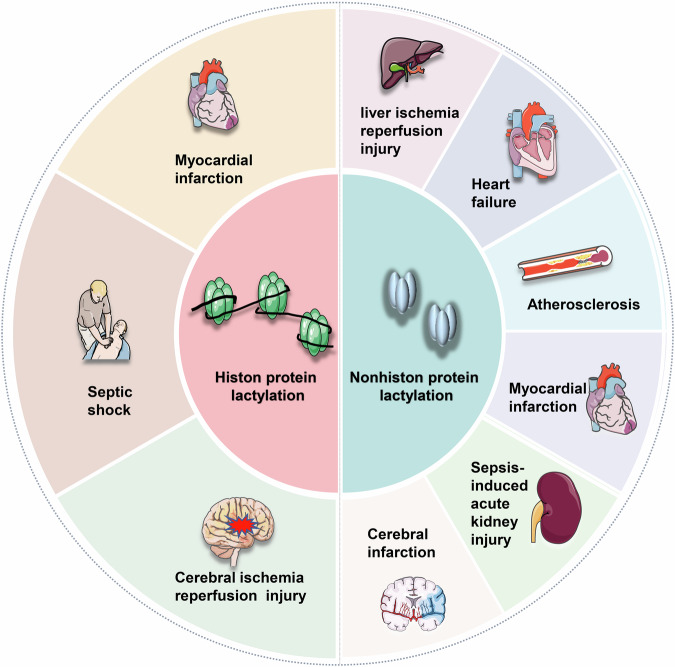
Lactylation in histone and nonhistone proteins: implications for ischemia-reperfusion injury. Lactylation in both histone and nonhistone proteins is a critical factor in several diseases linked to ischemia-reperfusion injury. This process significantly impacts organs and tissues such as the heart, liver, kidney, brain, and others, underscoring its importance in the pathophysiology of these conditions. Created with Figdraw.

In the context of IRI, diminished tissue perfusion during the ischemic phase leads to enhanced anaerobic glycolysis and subsequent lactate accumulation. Such metabolic shifts prompt the lactylation of both histone and nonhistone proteins, impacting the onset and progression of IRI. Future research endeavors will aim to further clarify the interplay between lactylation and IRI, building upon the existing body of evidence. Next, we will present a comprehensive analysis of the lactylation sites identified to be closely associated with IRI (Fig. 5, Table 2). This exploration will delve into the specific locations and their relevance to the pathophysiology of IRI, highlighting the critical role these sites play in the progression and manifestation of the injury.

**Table 2:** Lactylation sites associated with IRI and their principal effects.

lactylation	Sites	Species	Main effects	References
Histone lactylation	H3K18	Mouse	Histone lactylation boosts reparative gene activation post-myocardial infarction	[20]
	H3K18	Human	Lactylated histone H3K18 as a potential biomarker for the diagnosis and predicting the severity of septic shock	[37]
	H3K18	Rat	LDHA mediated the histone lactylation induced pyroptosis through targeting HMGB1 in the cerebral IRI	[38]
	H3K18	Rat	Mediating the protective effect of buyang huanwu decoction on cerebral IRI	[117]
Nonhistone lactylation	HMGB1	Mouse	Hepatocyte HSPA12A attenuates liver ischemia-reperfusion injury by suppressing glycolysis-induced HMGB1 lactylation	[83]
	Snail1	Mouse	Lactate promotes endothelial-to-mesenchymal transition via Snail1 lactylation after myocardial infarction	[82]
	K1897 of α-MHC	Human/Mouse	α-myosin heavy chain lactylation maintains sarcomeric structure and function and alleviates the development of heart failure	[62]
	K20 of Fis1	Mouse	PDHA1 hyperacetylation-mediated lactate overproduction promotes sepsis-induced acute kidney injury via Fis1 lactylation	[72]
	k271 of Mecp2	Mouse	Exercise-induced endothelial Mecp2 lactylation suppresses atherosclerosis via the Ereg/MAPK signaling pathway	[168]
	LCP1	Rat	Inhibition of the glycolysis prevents the cerebral infarction progression through decreasing the lactylation levels of LCP1	[118]
	METTL3	Mouse	Up-regulation of METTL3 lactylation enhanced the METTL3 protein stability and expression levels in Intracerebral hemorrhage progression	[112]
	YTHDF2	Mouse	Exercise training attenuates lactylation and mitigates myocardial IRI via YTHDF2 inhibition	[90]
	HSPA12A	Mouse	HSPA12A preserves aerobic glycolysis homeostasis and histone 3 lactylation in cardiomyocytes, thereby ameliorating myocardial IRI	[91]
	NCOA4	Mouse	Lactylation of NCOA4 enhances neuronal ferritinophagy and glycolysis following cerebral IRI	[113]
	ARF1	Mouse	Astrocytic LRP1 promotes mitochondrial transfer to neurons and alleviates cerebral IRI through suppression of ARF1 lactylation	[114]

*α-MHC* α-myosin heavy chain, *ARF1* ADP-ribosylation factor 1, *Ereg* epiregulin, *Fis1* mitochondrial fission 1 protein, *HMGB1* high mobility group box-1, *HSPA12A* heat shock protein A12A, *LCP1* lymphocyte cytosolic protein 1, *LDHA* lactate dehydrogenase A, *LRP1* low-density lipoprotein receptor-related protein 1, *MAPK* mitogen-activated protein kinase, *MECP2* methyl CpG binding protein 2, *METTL3* methyltransferase-like 3, *NCOA4* nuclear receptor coactivator 4, *PDHA1* pyruvate dehydrogenase E1 component subunit alpha, *YTHDF2* YTH N6-methyladenosine RNA-binding protein 2.

## MI

Annually, acute MI affects approximately 7 million individuals globally. Despite considerable advancements in therapeutic and diagnostic techniques, MI continues to be a predominant cause of mortality worldwide [84]. MI, commonly known as a heart attack, occurs due to plaque accumulation in the coronary arteries, leading to diminished blood flow and oxygen supply to the heart [85]. Research has elucidated that the pathogenesis and progression of MI are governed by a complex array of mechanisms. These include the generation of reactive oxygen species and pro-inflammatory mediators, activation of immune responses, stimulation of both angiogenesis and lymphangiogenesis, alterations in myocardial electrophysiology, and induction of cellular apoptosis [84].

Recent advancements in medical research suggest a significant link between epigenetic regulation and the pathogenesis of myocardial IRI, positing epigenetics as a potential novel therapeutic target for mitigating or preventing IRI [86]. Epigenetic modifications of histones involve a spectrum of processes such as methylation, acetylation, phosphorylation, ubiquitylation, sumoylation, among others [87]. Current investigations into myocardial IRI predominantly explore the dynamic and reversible nature of methylation and acetylation alterations [86]. For instance, studies suggest that the cardioprotective effect of H3K9 methylation may stem from its role in reducing inflammation by inhibiting the MAPK/NF-κB signaling pathway [86]. Furthermore, the inflammatory response following AMI is crucial in determining the extent of MI. Persistent pro-inflammatory reactions can lead to adverse post-MI left ventricular remodeling, highlighting inflammation as a vital therapeutic target to enhance outcomes post-AMI [88]. Interestingly, postconditioning with lactate-enriched blood has shown good protective effects on ST-segment elevation MI [89]. These findings emphasize the important role of histone epigenetic modifications and lactate-mediated inflammatory regulation in the pathogenesis and treatment of MI.

In 2022, Wang et al. elucidated that histone lactylation orchestrates the anti-inflammatory and pro-angiogenic dual functions of monocyte-macrophages. This process aids in reparative gene transcription, fostering a conducive environment for cardiac repair and enhancing cardiac function post-MI [20]. A subsequent study revealed a novel role of lactate in augmenting cardiac fibrosis and exacerbating cardiac dysfunction. This effect is mediated through the promotion of endothelial-to-mesenchymal transition, a key player in cardiac fibrosis, following MI [82]. Moreover, a clear association between lactylation modification and both heart failure and atherosclerosis has been established. The interaction between α-myosin heavy chain and Titin is essential for maintaining cardiac structure and contraction. The dynamic lactylation of α-myosin heavy chain is a critical determinant of cardiac integrity and function. A reduction in intracellular lactate levels, primarily due to excessive efflux and consumption by cardiomyocytes, contributes significantly to diminished lactylation at the K1897 site of α-myosin heavy chain during myocardial injury [62].

Furthermore, recent studies have elucidated significant effects of exercise on improving cardiac function and promoting metabolism. A key mechanism involves exercise training inhibiting YTHDF2 lactylation, thereby effectively preventing acute injury and pathological remodeling in myocardial IRI [90]. Additionally, HSPA12A has been shown to play a crucial role in myocardial protection by maintaining aerobic glycolysis homeostasis in cardiomyocytes and regulating histone 3 lactylation, thus alleviating myocardial IRI [91]. These findings underscore the complex and critical role of lactylation in myocardial IRI, acting as a double-edged sword with drastically different effects under various signaling pathways or intervention conditions. Further in-depth research is necessary to elucidate the specific mechanisms of lactylation in myocardial IRI.

Cardiomyocytes are remarkably sensitive to changes in energy metabolism, and epigenetic modifications play an instrumental role in the initiation and progression of MI. With ongoing research, it is anticipated that more lactylation sites, whether on histone or nonhistone proteins, will be identified in the context of MI.

## Liver IRI

IRI significantly contributes to liver damage during surgical procedures, such as hepatic resection and liver transplantation. It is a primary factor in graft dysfunction and post-transplantation liver failure [4]. Liver IRI involves a complex interplay of cellular and molecular mechanisms, leading to an exacerbated inflammatory response [92]. Liver-resident macrophages and recruited cells produce pro-inflammatory signals, including reactive oxygen species, cytokines, chemokines, proteases, and lipid mediators. These signals trigger the inflammasome, intensifying the inflammatory response and accelerating hepatocyte necrosis and apoptosis [4, 92, 93].

PTMs profoundly affect almost all proteins, playing a vital role in preserving the functionality and diversity of the proteome. The role of PTMs in the onset and progression of liver IRI has been substantiated by numerous studies, with ubiquitination standing out as a key area of research [94, 95]. For example, the NF-κB-mediated inflammatory response is crucial in liver IRI, and the autopolyubiquitination of K63-linked tumor necrosis factor receptor-associated factor 6 is essential for NF-κB activation. OTU domain protein 4, acting as a deubiquitinating enzyme, interacts with tumor necrosis factor receptor-associated factor 6 to reduce its K63 autopolyubiquitination, thereby mitigating liver ischemia-reperfusion injury [94].

In addition to ubiquitination, histone-mediated PTMs are also implicated in liver IRI. Notably, the SIRT1 can attenuate liver IRI in mice. This mitigation is achieved by inhibiting the XBP1/NLRP3 inflammatory pathway through miR-182 activation, while SIRT6 offers protection against liver IRI by repressing apoptosis and autophagy-related cell death [96, 97]. Liver IRI is characterized by two interlinked phases: ischemia-induced hepatocyte injury, leading to the release of damage-associated molecular patterns, and subsequent recruitment of immune cells, initiating an inflammatory cascade that exacerbates hepatocyte damage. HMGB1 is a key damage-associated molecular patterns in this process. Research has shown that HSPA12A, an unconventional member of the HSP70 family, inhibits HMGB1 lactylation and secretion from hepatocytes, mediated by glycolysis. This inhibition curtails macrophage chemotaxis and inflammatory activation, ultimately offering protection against liver IRI [83]. Lactylation significantly increases HMGB1 release from hepatocytes, a key component of damage-associated molecular patterns, actively mediating subsequent liver IRI. Moreover, HMGB1 serves as an indispensable intermediate bridge in HSPA12A’s protection against liver IRI.

These observations strongly suggest that PTMs of both histones and non-histones may play pivotal roles in liver IRI pathogenesis. While research on the connection between lactylation and liver IRI is still in its infancy, the liver’s unique position as the core organ of lactate metabolism suggests that future advancements will likely reveal more lactate-mediated lactylation sites closely related to liver IRI.

## Septic shock

The study of septic shock remains a critical area of interest, owing to its propensity for inducing severe complications and mortality. Frequently, the host’s pro-inflammatory response, aimed at eliminating the invading pathogens, may inadvertently inflict collateral tissue damage. This is especially evident during episodes of severe sepsis. In contrast, the anti-inflammatory response is crucial in mitigating both localized and systemic tissue injury. Nevertheless, its overactivation or inappropriate engagement can increase the susceptibility to secondary infections. Furthermore, factors such as innate immune responses, coagulopathy, the modulation of the anti-inflammatory/pro-inflammatory equilibrium, and immunosuppression, collectively play a role in the onset and progression of septic shock, potentially leading to organ dysfunction [98].

The pathogenesis of septic shock is complex and multi-dimensional. Studies have shown that PTMs such as ubiquitination and acetylation are implicated in the initiation and progression of this condition [99, 100]. For instance, a key element in the innate immune response is the inflammation mediated by Toll-like receptors. The E3 ligase RNF99 plays a critical role by interacting with and targeting TAK1-binding protein 2 for degradation. TAK1-binding protein 2 is a regulatory protein of the kinase TAK1, and its degradation occurs via the K48-linked ubiquitin-proteasomal pathway at lysine 611. This interaction is instrumental in modulating the Toll-like receptors-mediated inflammatory response [99]. Additionally, circulating histones such as H2B, H3, and citrullinated H3 have been identified as valuable biomarkers for the early diagnosis of sepsis and the assessment of its severity [101, 102].

Septic shock, a systemic disease, can profoundly affect multiple organ systems. Recent studies have highlighted the role of SIRT3 downregulation in mediating the hyperacetylation and subsequent inactivation of the pyruvate dehydrogenase E1 component subunit alpha. This alteration leads to excessive lactate production in renal tubular epithelial cells, contributing to the pathogenesis of septic shock. The resultant increase in lactate levels can induce lactylation of the mitochondrial fission 1 protein, exacerbating sepsis-induced acute kidney injury. Conversely, reducing lactate levels and mitochondrial fission 1 protein lactylation may attenuate this injury [72]. Further research has identified lactylation as a novel PTM in peripheral blood monocytes, observed in both healthy individuals and critically ill patients. Notably, patients with septic shock exhibit a significantly higher relative density of this modification. Lactylation at H3K18 is believed to regulate the expression of inflammatory cytokines and promote the overexpression of arginase-1, thereby enhancing the anti-inflammatory response of macrophages during sepsis [37].

These findings underscore the critical role of histones and their PTMs in both the detection and progression of septic shock. This is further supported by the strong correlation between lactate levels, including their temporal changes, and the morbidity and mortality associated with sepsis, as well as tissue hypoxia and hypoperfusion [103]. These research results indicate that lactylation has potential as a treatment target for sepsis-induced injury. Given that sepsis is a systemic injury disease, the high expression of certain lactylation sites may serve as diagnostic and predictive indicators, expanding the application scope of lactylation in sepsis research. Consequently, future research endeavors in septic shock may benefit from a focus on lactate and its associated lactylation processes.

## Cerebral IRI

Stroke, ranking as the second leading cause of death worldwide, results in a spectrum of severe cerebral disorders, leading to mortality and varying degrees of impairment [104, 105]. Reperfusion of ischemic brain tissue exacerbates oxidative stress and triggers an increased release of pro-inflammatory cytokines. This initiates a series of pathological cascades, culminating in apoptosis, blood-brain barrier disruption, cerebral edema, and hemorrhagic transformation [105]. The progression of cerebral IRI is multifactorial, involving activation of microglia and astrocytes, immune cell infiltration, platelet activation, and various cell death pathways such as apoptosis, necrosis, and ferroptosis. Among these, the inflammatory cascade is particularly notable as a central mechanism exacerbating cerebral IRI [105, 106].

Studies have shown that following focal and global cerebral ischemia, there is an elevation in ubiquitin-protein conjugates within the brain. Post-ischemic ubiquitination is rapidly induced upon reperfusion and is transiently observed in potentially viable neurons within the peri-infarct area [107]. Recent advances in molecular biology reveal that gene expression can be regulated by PTMs without altering the genetic code’s chemical structure. In this context, HDACs play a critical role. These enzymes repress gene expression by removing acetyl groups from lysine residues on histones, leading to chromatin compaction. The pharmacological inhibition of HDACs has been explored for activating protective genes, and their role in the mechanism of cerebral IRI has been studied [108]. Moreover, preclinical evidence suggests that HDAC inhibitors could be a promising and cost-effective therapeutic strategy for enhancing neurological and cardiac outcomes post-IRI. This therapeutic potential is primarily achieved by promoting angiogenesis, neurogenesis, and stem cell migration, which collectively contribute to a marked reduction in infarct volume and enhanced functional recovery in experimental models of cerebral ischemia [109, 110].

A recent proteomic study revealed that in a rat model of acute ischemic stroke, lactylation of cortical proteins was modified at 1003 sites across 469 proteins [111]. Additionally, research indicated that treating N2a cells with oxygen-glucose deprivation/reoxygenation upregulated HMGB1 and lactate expression and increased histone lactylation levels. Lactate dehydrogenase A modulates pyroptosis induced by histone lactylation in cerebral ischemia through HMGB1. Inhibiting lactate dehydrogenase A expression has been shown to reverse this pyroptosis [38]. Interestingly, lactylation’s role extends beyond cerebral ischemic conditions. It also appears to be associated with cerebral hemorrhagic diseases. Intracerebral hemorrhage, which results from increased vascular fragility and rupture in the non-traumatic brain parenchyma, has been strongly linked to ferroptosis. A study demonstrated that lactylation of METTL3 increased in heme-treated PC12 cells. This enhancement led to greater protein stability and elevated METTL3 expression, which in turn upregulated the m6A level and mRNA expression of the transferrin receptor, further inducing ferroptosis in the PC12 cells [112].

Recent findings reveal that various molecules and pathways, including nuclear receptor coactivator 4, astrocyte-derived lactate, low-density lipoprotein receptor-related protein 1, and cGAS signaling, can influence cerebral IRI progression through unique lactylation mechanisms or by directly regulating overall lactylation levels [113–116]. Notably, lactylation also acts as a key mediator for some drugs in cerebral IRI protection, such as Buyang Huanwu Decoction, which effectively inhibits cerebral IRI deterioration by suppressing extensive lysine lactylation and specific H3K18 lactylation [117].

These findings highlight lactylation’s crucial role in cerebral IRI, demonstrating distinct bipolar characteristics with potential benefits and non-negligible drawbacks. Furthermore, lactylation shows promise as a key intermediate mediator for certain drugs to achieve cerebral IRI protection. Given that glucose metabolism is the brain’s primary energy source, an adequate blood supply is essential for sustaining brain function. During cerebral ischemia, the brain predominantly relies on anaerobic glycolysis for energy, resulting in the accumulation of lactate, which mediates an increase in lactylation. Notably, there is evident reciprocal regulation between lactylation and inflammation. This suggests that targeting lactylation could provide promising intervention strategies for cerebral IRI.

## Conclusions and perspectives

Adequate blood supply is essential for sustaining normal bodily functions, and IRI has been shown to play a pivotal role in the onset and progression of various diseases. These include myocardial infarction, hepatic IRI, septic shock, and cerebral IRI, as evidenced in studies [37, 83, 86, 118]. During tissue ischemia, cellular energy metabolism undergoes a shift, with glycolysis becoming the dominant source of energy. This metabolic shift can lead to increased lactate production and accumulation, consequently resulting in increased lactylation.

Lactylation, a PTM first identified by Professor Zhao Yingming’s research team in 2019, has emerged as a key focus in PTM research [16]. Lactylation predominantly involves modifications on lysine residues of histone or nonhistone proteins, triggered by lactate accumulation. This modification has been linked to the development and progression of various diseases, such as melanoma, myocardial infarction, hepatocellular carcinoma, Alzheimer’s disease, and nonalcoholic fatty liver disease [18–24]. Additionally, research has revealed a complex interplay between lactylation and inflammation [25–28]. These regulatory mechanisms extend beyond energy metabolism to encompass broader biological significance. Recent investigations have demonstrated that during IRI, lactate accumulation and subsequent lactylation effects function as critical molecular bridges between metabolic stress and cellular responses [37, 83, 86, 118]. The interaction between lactylation modifications and inflammatory regulatory mechanisms exhibits complex bidirectional characteristics. Specifically, lactylation directly modulates the activity of key inflammatory mediators, including NF-κB and STAT proteins, enabling precise regulation of inflammatory responses [55, 67]. Additionally, lactylation-mediated chromatin modifications establish specific epigenetic environments that modulate inflammatory gene expression in a context-dependent manner [16, 67, 68]. Furthermore, lactylation of metabolic enzymes, particularly PKM2 and IDH3G, significantly influences cellular energy metabolism, thereby affecting immune cell function and inflammatory responses [56, 119].

The Warburg effect, pivotal in advancing our understanding of glycolysis, has clarified that lactate production can occur under both aerobic and anaerobic conditions. This phenomenon underscores the need for further investigation into the regulatory roles of enzymes, such as HDACs, in histone PTMs. A critical aspect of this research focuses on the balance between histone acetylation and lactylation. Studies have shown that histone lactylation and acetylation can compete for the epigenetic modification of lysine residues, thereby influencing the levels of lactate and acetyl-CoA. Further research is essential to determine whether pyruvate, at the glycolytic pathway’s terminus, preferentially contributes to lactate or acetyl-CoA synthesis, impacting subsequent cellular developmental processes [60]. Moreover, lactylation and acetylation often share writer and eraser mechanisms, playing antagonistic roles in various diseases. Studies have shown that under lactate-deficient conditions, H3K18 lactylation is significantly reduced, correlating with changes in gene expression patterns, while H3K27 acetylation shows minimal correlation. These findings suggest that lactate may be more inclined to regulate early embryonic development by affecting H3K18 lactylation rather than H3K27 acetylation [120].

Moreover, in the context of IRI, substantial evidence indicates significant crosstalk between lactylation and other PTMs. During sepsis, macrophages utilize monocarboxylate transporters for extracellular lactate uptake, promoting HMGB1 lactylation through p300/CBP-dependent mechanisms. Concurrent with this process, lactate induces HMGB1 acetylation through dual pathways: Hippo/YAP-mediated SIRT1 and β-arrestin2 inhibition, and G-protein-coupled receptor 81 (GPR81)-mediated recruitment of p300/CBP acetyltransferase. The resultant lactylated/acetylated HMGB1, released via macrophage-derived exosomes, subsequently enhances endothelial cell permeability. Notably, in vivo reduction of lactate production or inhibition of GPR81-mediated signaling pathways significantly decreases circulating exosomal HMGB1 levels, thereby improving survival outcomes in polymicrobial sepsis [28]. These observations demonstrate that individual proteins can undergo simultaneous lactylation and acetylation modifications, potentially generating synergistic effects. Although comprehensive understanding of the competitive mechanisms between these PTMs requires further investigation, current evidence strongly suggests intimate regulatory connections between lactylation and other PTMs, particularly acetylation. Future research is expected to reveal increasingly complex and diverse interactions between lactylation and various PTMs networks. These findings emphasize the close connection between lactylation and other PTMs, particularly acetylation. As research deepens, we have reason to believe that more evidence will reveal the complex and diverse interactions between lactylation and other PTMs.

Notably, the lactylation of the nonhistone protein YY1 in microglia has been identified as a key factor in retinal neovascularization, primarily through upregulating FGF2 expression [121]. Concurrently, studies have shown that evodiamine effectively inhibits lactate-induced angiogenesis in prostate cancer cells. This inhibition occurs by modulating histone lactylation and HIF1A expression, which consequently enhances Sema3A transcription and suppresses PD-L1 expression [122]. These results clearly reveal that lactylation also has significant regulatory effects in angiogenesis, expanding its application prospects in IRI to early angiogenesis and blood supply restoration. These discoveries enhance our comprehensive understanding of the interaction mechanisms between lactylation and IRI.

It is worth noting that the interactions between different lactylation sites in histones are equally thought-provoking. To date, numerous lactylation sites have been identified on histones, covering almost all five histone subtypes, with H3K18 being particularly well-studied. In various diseases, multiple sites are modified simultaneously [123]. Recent studies suggest synergistic effects of H3K18 and H3K9 lactylation in regulating CD8 T-cell effector function [124], and H3K14 and H3K9 lactylation in promoting calcification in calcific aortic valve disease [125]. Therefore, it is particularly important to deeply explore the differences and connections between different modification sites on histones. Furthermore, lactylation also regulates various enzyme activities or acts as downstream pathways of enzymes, including aldehyde dehydrogenase 1A3, lactate dehydrogenase A and B, and HDAC2 [126–129]. These pathways further expand the modes and ranges of lactylation’s effects in different diseases, emphasizing the important role of lactylation in regulating disease progression. However, current investigations of histone lactylation modifications have predominantly focused on specific modification sites, particularly H3K18 [20, 37, 65], while studies examining modifications at other histone sites remain limited. Although this site-specific investigative approach has provided valuable mechanistic insights, it potentially constrains comprehensive understanding of the global effects of histone lactylation modifications. Moreover, the temporal dynamics of histone lactylation modifications necessitate more extensive investigation to fully elucidate their biological significance.

Overall, in the context of IRI, both anaerobic glycolysis during ischemia and the exacerbation of cellular functional damage during reperfusion are instrumental in lactate production and accumulation. This lactate subsequently induces histone or nonhistone lactylation, playing a pivotal role in the progression of IRI. Despite these insights, several aspects of lactylation remain elusive. These include the complex equilibrium of various PTMs on histone lysine residues, the potential interplay between histone and nonhistone lactylation and their respective impacts on cellular function, and their distinct roles in the pathophysiology of IRI. Notably, recent studies have unveiled significant interactions between histone and nonhistone lactylation modifications. These distinct PTMs share several crucial regulatory enzymes, including P300 as a writer enzyme [16, 18, 121] and HDACs/SIRTs functioning as erasers [18, 59, 78]. The recent characterization of novel acetyltransferases AARS1 and AARS2 provides additional evidence supporting coordinated regulatory mechanisms between these PTMs [79–81]. However, the precise molecular interactions between these modification types and their detailed coordination mechanisms in cellular responses remain incompletely understood, particularly within the context of IRI. Enhanced understanding of these intricate interactions may reveal novel therapeutic strategies targeting lactylation modifications. Further investigation of the molecular mechanisms governing lactylation writers and erasers is essential for developing a comprehensive understanding of this PTM system and elucidating complex interactions among various PTMs. Notably, the regulatory network between lactylation and acetylation modifications likely extends beyond currently identified factors such as HDACs, histone acetyltransferase p300, and AARS1/2, suggesting the potential existence of additional regulatory components awaiting discovery.

Moreover, an in-depth exploration of inter-organ communication in response to IRI represents a highly promising research direction. Of particular interest is the rapid interaction between intestinal and cerebral IRI mediated by neural circuits. Notably, cerebral IRI has been shown to rapidly induce secondary intestinal IRI, a finding that has opened up novel avenues for investigation in this field [130]. Advances in technological fields, particularly in proteomics, are expected to facilitate the increased identification of lactylation sites, especially nonhistone ones. This progress will likely open new avenues for research and provide innovative strategies for intervention. Such developments are anticipated to significantly enhance our understanding of disease pathogenesis and inform the development of therapeutic approaches for related conditions.
